# The Effects of the Principal Arts, Trades, and Professions, and of Civic States and Habits, on Health and Longevity

**Published:** 1831-04-01

**Authors:** 


**THE EFFECTS OF THE PRINCIPAL ARTS, TRADES, AND PROFESSIONS, AND OF THE CIVIC STATES AND HABITS OF LIVING, ON HEALTH AND LONGEVITY. By *C. Turner Thackrah.* Octavo, pp. 126. Longmans, London, 1831.**

DR. THACKRAH, from his residence as a medical practitioner, in the great manufacturing city of Leeds, has had ample opportunities of observing the general and peculiar effects of trades and professions on the human constitution. Our author has followed the path of Ramazini, and has produced a volume replete with useful observations and sensible remarks, interesting to all medical practitioners, but especially to those who reside in manufacturing towns.

Dr. T. asks these two questions—do the fifty thousand men employed in the manufactories of Leeds enjoy that vigour of body which is ever a direct good, and without which all other advantages are worthless? Do they live as long as the agriculturalists? The unhealthy appearance of the population affords an unequivocal reply to the first question. The bills of mortality answer in the negative to the second. For the convenience of inquiry our author divides the inhabitants of Leeds into four classes—the operatives—dealers—master-manufacturers and merchants—professional men. Dr. T. commences with those operatives that live a good deal in the open air, and who "come nearest the perfection of the physical state."

At the head of this list stand the BUTCHERS. These are much in the open air, take active exercise, and eat fresh animal food at least twice a day. They are plump and rosy—cheerful and good-natured—nor does their bloody occupation render them savage, as theorists pretend, or as the English law presumes. They live in easier circumstances than most other operatives, for meat is always in requisition, whether the times be good or bad. They are subject to few ailments—and those chiefly from plethora. It is very rare to see any of them phthisical. Yet they are not remarkable for longevity—they are even rather shorter lived than people less healthy. In fact, they live too well to live very long.

FISHMONGERS, who bring fish from the coast, are much exposed to the weather, and are generally temperate, hardy, and healthy. Not so the town retailers of fish. They are addicted to dram-drinking, and are sickly and short-lived.

CART-DRIVERS, labourers in husbandry, sand-leaders, bricklayers, and such people, would be healthy if they were well fed; but this, unfortunately, is not the case. The bricklayers, though so much exposed to wet and cold, are seldom affected by rheumatism, catarrhs, or pneumonia.

CHAISE-DRIVERS and postillions would be very healthy, did they not drink so much spirit. Postillions, indeed, from their postures, exercise some parts of the body more than others, and are liable, for obvious reasons, to curvatures of the spine.

"The atmospheric vicissitudes to which all drivers are exposed, are thought to produce rheumatism and inflammation of the lungs. I conceive, however, that these diseases would rarely occur to abstemious men It is intemperance which gives the susceptibility to such maladies; and it is intemperance which produces much greater, the fatal affections which we have just mentioned. I scarcely need add, that the whole class is short-lived. They generally die before they reach the age of 50. Among all the Leeds men, we could find only three individuals who are old, and two of these have the character of great temperance." 12.

Passing over some subordinate classes of operatives, we come to those who carry on their avocations in confined or impure air. The air of Leeds is nearly as much impregnated with carbon as the air of London. This state of atmosphere affects the inhabitants. Their complexion is pallid—"and the tongue shews that digestion is disordered and imperfect." Dr. T. thinks that not a tenth part of the inhabitants of Leeds enjoy full health. The lungs suffer much less from this sooty atmosphere than might be expected. Tailors, from their accumulation in crowded rooms, and the want of air and exercise are very unhealthy. Disorders of the stomach and bowels are general, and often obstinate. Pulmonary consumption is also frequent. We see no plump and rosy tailors—none of fine form and strong muscle. The spine is generally curved. We are rather surprized at this—for the muscles of the spine are more worked than any other muscles of the body except those of the right arm. The tailor, like other men whose physical energies are depressed, often seeks the baneful comfort of ale and ardent spirit. The following suggestion, we fear, will not be adopted.

"The position of the tailor might be amended. He now sits cross-legged on a board; because in the ordinary sitting posture he could not hold a heavy piece of cloth high enough for his eyes to direct his needle. Let a hole be made in the board of the circumference of his body, and let his seat be placed below it. The eyes and the hands will then be sufficiently near his work ; his spine will not be unnaturally bent, and his chest and abdomen will be free. I am aware that old workmen will be unwilling to regard this or similar suggestions; for every man is formed to his habits. If however masters and medical men would urge an alteration, and if especially boys apprenticed to the trade were taught to work in the posture recommended, tailors would assuredly become much more healthy. The practice of drinking might also be easily reduced, if masters discharged from their employ every man who absented himself a day without proper cause." 18.

We must pass over numerous descriptions of workmen, only selecting a class here and there. We find that CURRIERS and LEATHER-DRESSERS are generally very healthy and long-lived, though subject to the bent posture in the process of shaving. This affects the head. The smell of the leather is not injurious. Compositors are not healthy. The standing posture, Dr. T. observes, injures the digestive organs. Consumption is frequent among them ; and we seldom can find any who have attained 50 years of age.

"SMITHS have an employment remarkably conducive to muscular power. The use of the large hammer powerfully excites all the muscles, and especially those of the arms, throwing on them a large supply of blood, and consequently producing their enlargement. Exertion like this, moreover, has a considerable effect on the circulation in general, and the functions with which it is connected. For youths of strong constitution, no labour is better than that of the smith. For those, however, naturally delicate, the exertion is too great, and young men of scrofulous constitution are particularly liable to sink under the employ. Smiths are subjected to high temperature, and frequent changes of temperature, but with no obvious injury. They are rarely affected with rheumatism and catarrh. The employ subjects the eye to the annoyance of smoke, and to excitement from the glow of the heated iron. But our examination of the smiths in this neighbourhood does not prove them subject to ophthalmia; nor does it show that vision is impaired by the excitement of the retina. When smiths are ill, the cause is most frequently intemperance. They do not however arrive at great age. We could hear of but one old smith in the town of Leeds." 26.

HOUSE-SERVANTS, from confinement in a smoky town, are seldom healthy. Girls from the country soon lose their ruddy complexion, and suffer more than the natives of the town. COLLIERS have considerable muscular exertion, chiefly in the sitting or kneeling posture, and with the body bent to a very acute angle. They work in an unnatural atmosphere, and with artificial light, exposed to changes of air, and often to wet feet. Sometimes the perspiration is excessive. They are generally spare men, with curved spines and bowed legs. The skin, of course, is loaded with dirt; and when this is removed, the complexion appears sallow and unhealthy. Their eyes appear small, affected with chronic inflammation, and intolerant of light. Colliers are subject to disorders of the head, muscular pains, rheumatism, and asthma.

The author next comes to those employments which produce dust, odour, or gaseous exhalations. He divides these into three classes, namely, where the above substances are not apparently noxious—where they appear to be beneficial—and where they are decidedly injurious.

1. In the first class he places workers in wool and leather, butchers, provision-dealers, cooks, starch-makers, rectifiers of spirit, bricklayers, plasterers, white-washers, wool-sorters, turners, tobacconists, snuff-makers, &c.

All these are exposed to extraneous substances, tangible or gaseous, dispersed through the atmospheres which they breathe, but without any appreciable bad consequences to their health. We shall introduce the following note on smoking and snuff-taking, as a specimen of our author's manner.

"It is amusing to read the opinions on the *use* of tobacco, held a century or two ago by some eminent physicians. Bonetus quotes from Augustinus Thonerus, that a certain court-physician, not contented with smoking in the day, would have a lamp with wax-candles and pipes suspended to his bed. Dying a year after, and his head being opened, 'res stupenda!' the whole brain was so dried that it scarcely exceeded the magnitude of a nut. Pauvius mentions the dissection of a robust and healthy young man, whose brain was tinged with a black smut. He accounted for the fact when he found the man had been a confirmed tobacco-smoker. Mention is made of a military officer aged 73, who had been addicted to tobacco from childhood. He smoked and chewed incessantly. On dissection, Krantrius found adherent to the cranium much of a dense substance, resembling chimney soot in appearance as well as taste. All the mischief was attributed to the tobacco. Bonetus, Diemerbroeck, and others, thought it necessary to counteract this opinion by the relation of a number of negative cases, as amusing in their details as those I have quoted.

We are often asked if the use of tobacco is injurious? Viewing the question in the abstract, we should answer, Yes. To a person in full health, nothing is required but pure air, food, and drink: every thing else is superfluous, and consequently oppressive to the constitution. A narcotic substance must be more than oppressive, because it makes a direct attack on the nervous system. It affects the stomach and the brain. But viewing man as the creature of civilization, subjected hourly to excitement foreign to his nature, and injurious to his health, narcotics, by allaying nervous excitability, may, in certain circumstances and constitutions, be really useful. We would not therefore deprive the smoker of his consolation, but we would keep the practice from excess. We would guard especially against that unnecessary potation, to which the practice so frequently leads. Drinking is a great and positive evil; smoking is at best but a slight good. If the two must be associated, banish them as decidedly inimical to health and reason. Smoking can never be proper before the middle period of life. For young men to parade the streets in the evening, with cigars in their mouths, is either affectation or something worse." 33.

The use of tobacco, in fact, is like the use of wine or other stimulant. It is only in excess that the injury lies. In some countries too, where malaria, excessive cold, or inordinate moisture prevails, tobacco, we believe, is positively useful. It is in such countries and localities that we see the cigar most in use—and we should not always despise vulgar habits and customs. They have generally a basis in utility.

We now proceed to those employments where extraneous admixtures in the atmosphere are supposed to be beneficial. The following sentiment contains good sense.

"To assert the existence of such an effect may seem a contradiction to the statement before made, that whatever alters the natural constitution of the atmosphere, must be proportionately injurious. But it should be remembered that injurious agents sometimes counteract each other. Medicine is in itself an evil. Remedies often induce unnatural states, but these states supersede others much more serious and permanent. To man in a perfectly healthy condition, no substance arising from manufacture can be useful; but men living in a large town, and with the habits of civil life, are generally unhealthy; and hence certain vapours or other substances may be decidedly beneficial in exciting their languid powers, or correcting the disposition to disease." 33.

RAPE AND MUSTARD CRUSHERS inhale a peculiar odour from the seeds which they grind. "This seems to act as a stimulant on the nervous and circulatory system; for men fresh to the employ find their appetite and vigour increased." The heat of the room is considerable—often 80° in Summer; and though addicted to intemperance, the men employed in oil-mills are generally healthy.

BRUSH-MAKERS inhale a good deal of the odour of pitch, which appears to have a sanative effect in bronchial affections, chronic catarrh, and asthma. "The workmen are generally free from disease."

GROOMS AND HOSTLERS daily inhale a large quantity of ammoniacal gas generated in the stables. "This appears beneficial rather than injurious."

"GLUE AND SIZE BOILERS are exposed to strong putrid and ammoniacal exhalations from the decomposition of animal refuse. The stench of the boiling and drying rooms is indeed well known to be highly offensive, even to the neighbourhood. Yet the men declare it agrees well with them—nay, many assert that on entering this employ, they experienced a great increase of appetite and health. All the glue and size boilers we saw, were remarkably fresh-looking and robust. Though exposed to frequent and considerable changes of temperature, to sudden changes also from an atmosphere of hot vapour to the dry cold air, they are not subject to rheumatism, pulmonary inflammation or catarrh. The only complaints we could hear of, were occasional pains in the loins and limbs, attributable to posture and exertion." 35.

TALLOW-CHANDLERS, who are exposed to an offensive animal odour, enjoy good health, and attain a considerable age. It was remarked that, during the plague in London, this class of men suffered much less than others.

TANNERS, it is well known, are subject to disagreeable odours. They work in an atmosphere largely impregnated with the vapour of putrifying skins; and this, combined with the smell of lime in one place, and of tan in another. They are exposed constantly to wet and cold. Their feet are scarcely ever dry.

"Yet they are remarkably robust; the countenance florid; and disease almost unknown. Tanners are said to be exempt from consumption; and the subject has of late been repeatedly discussed in one of the medical societies of London. We have carefully inquired at several tan-yards, and could not hear of a single example of this formidable disease. We do not find *old* men actually in the employ; and the reason assigned is, not the decline of health, but the inferiority of men past middle age, in undergoing the labour of the process. Persons however in advanced life, yet healthy, are found in other occupations, who have before been for many years in the tan-yards, and have not apparently suffered from the long-continued exposure to their offensive odour. Hence we may infer that this employ, while it invigorates the constitution in youth and middle age, does not sensibly shorten life; does not, in other words, give temporary health at the expense of premature decline.

Ramazzini tells us that at Padua the tan-yards were permitted only in the suburbs. Here also, as the stench would be considered a nuisance, tan-yards are at the outskirts. As a matter of medical police, however, we see no occasion for their exclusion from the town." 36.

The 3d class includes those employments which are actually deleterious to health—and unfortunately they are numerous.

CORN MILLERS are placed first on the list by the author. They breathe an atmosphere loaded with the particles of flour, and suffer considerably.

The mills indeed are exposed to the air—the men are not crowded—and the labour is good. Yet millers are generally pale and sickly, with defective appetites, and indigestion. Many of them are annoyed by morning cough and expectoration, and some are asthmatic at an early period of life. The average quantity of air thrown out at expiration, among ten men that were examined, was seven pints. The evils of the employ, the author thinks, might be much diminished by exercise in the open air; but in this as in other employments, the men are very careless of their health. They think nothing about injurious agents till their health is destroyed. Maltsters appear to be still worse off than the millers.

"Workers in Flax, from their number and the effect of their employ, deserve particular attention. In the Flax-mills, all the departments, with the exception of the spinning and reeling, produce dust. The roving-rooms have a little, and the dry-house has a varying quantity. The carding-rooms are also dusty; but the worst department is certainly the heckling. This, in some mills, is carried on by hand, and in such the rooms are greatly clouded. In other mills, where the process is effected by machinery, the quantity of dust is considerably less. Still, however, it is such that a visiter cannot remain many minutes without being sensible of its effects on respiration. Children and a few overlookers are here the operatives; but in the old mode, I believe, men only are employed. Though attention is generally paid to ventilation, and the rooms for the several departments are spacious, they are not sufficiently lofty. A suffocating sensation is also often produced by the tubes which convey steam for heating the rooms. Persons in the dusty departments are generally unhealthy. They are subject to indigestion, morning vomiting, chronic inflammation of the bronchial membrane, inflammation of the lungs, and pulmonary consumption. The dust, largely inhaled in respiration, irritates the air-tube, produces at length organic disease of its membrane, or of the lungs themselves, and often excites the development of tubercles in constitutions predisposed to consumption. There is little doubt that a considerable quantity is also swallowed with the saliva, and deranges, in a greater or less degree, the functions of the stomach." 40.

Very few old persons are found in any of the departments of the flax-mills. Of 1079 persons exployed in one large establishment, only nine could be found above the age of 50 years.

We must now pass over a great portion of the work, since the subjects are so multifarious as to render it impossible to preserve any thing like a chain of analysis. There are some observations, however, on the effects of long-continued heat, alternated with cold, which are deserving of notice.

"In reference to catarrh in general, I might urge, were it part of my subject, that this disorder is often prevalent when the atmosphere is comparatively mild, and when there have been no great variations of temperature. I might also urge the fact of bed-ridden persons being attacked with catarrh, when no change has been made in the temperature or ventilation of their rooms. I might also repeat the general remark, made in a former page, that those persons who take most care to avoid atmospheric transitions, are not least, and are often indeed most, liable to catarrh and bronchitis; while those, on the contrary, who expose themselves most freely, are least and most rarely affected.

The inhabitants of this country are brought up from childhood in the fear of taking cold. It is the bugbear of our youth; it haunts us through life. Few have any distinct ideas of the nature of this frigorific agency: few have fairly examined their experience: and few indeed are aware of the fallacies of experience; scarcely any have made either direct experiment or close observation.

Yet all speak with decision on the subject. Hence workmen, when interrogated on the effects of change of temperature in producing coughs and catarrhs, commonly reply from their prejudices, rather than their observation. 'We *must* take cold: we are always in heats and cools.' But on cross-questioning them, I have not been able to satisfy myself, that men subjected to the greatest changes, are especially liable to catarrhs and bronchial inflammation. We do not hear more coughs in the Foundries, Dry-houses, or Press-shops, than in other places. The men, it is clear, are not particularly subject to consumption; and if the heat or transitions of their employ frequently excited bronchial inflammation, a pathologist would surely expect the prevalence of that fatal disease." 80.

Nothing can be more true than that constant and regular exposure to atmospheric vicissitudes is the only preservative against their effects. The more quickly repeated these vicissitudes are, the less danger there is from them. It is where the intervals between the vicissitudes are long, and the vicissitude great in range, as in Italy and many other countries, that the detriment is considerable, because the alternations of temperature are not rapid enough to accustom the human machine to their occurrence.*

Our author next proceeds to the second great class, beginning with a very numerous one, shop-keepers and dealers, who live a sedentary life, but are not particularly prone to disease. The want of pure air is the chief cause of their ailments. Commercial travellers have great advantages in this respect; but they destroy their constitutions by intemperance.

MERCHANTS AND MANUFACTURERS. These, as far as Leeds is concerned, spend most part of their time in the counting-house or the mill, subjected not only to impure air, but occasionally to dust and effluvium from the manufacture. These evils are occasionally diminished by sleeping in the country, or at a distance from the manufactory or warehouse; but generally these people keep near to the seat of their business—eat their meals in a hurried manner, as if they every moment expected to be summoned by a stage-coach driver—and take but little exercise in the open air. Other mercantile men, though they spend more time at their meals, are engaged in close thought; so that they taste neither their food nor their drink. The nervous energy required by the stomach for digestion, is abstracted by the mind. They think when they ought to eat. The animal operations are sacrificed to calculation, speculation, and commercial arrangement. The state of the bowels is also neglected, and foundation for serious disease laid.

"But of all agents of disease and decay, the most important is *anxiety of mind*. When we walk the streets of large commercial towns, we must be struck with the hurried gait and care-worn features of the well-dressed passengers. Some young men indeed we may remark, with countenances possessing natural cheerfulness and colour, but these appearances rarely survive the age of manhood. Cuvier closes an eloquent description of animal existence and change, with the conclusion that 'Life is a state of Force.' What he would urge in a physical view, we may more strongly urge in a moral. Civilization has changed our character of mind as well as of body. We live in a state of unnatural excitement; unnatural, because it is partial, irregular, and excessive. Our mus-

* See an examination of this subject in Dr. Johnson's "Change of Air," Part the Third, on the Climate of Italy.

cles waste for *want* of action: our nervous system is worn out by *excess* of action. Vital energy is drawn from the operations for which nature designed it, and devoted to operations which nature never contemplated. Though we can scarcely adopt the doctrine of a foreign philosopher, 'That a thinking man is a depraved animal,'—we may without hesitation affirm, that inordinate application of mind, the cares, anxieties, and disappointments of commercial life, impair the physical powers, and induce premature decay. The various disorders, generally known under the name of indigestion, disorders dependent on a want of circulation of blood through the bowels, biliary derangements, constipation, and headache, are well known to be the general attendants on trade, closely pursued. Indeed in almost every individual, this absorbing principle produces one or other of the various maladies to which I have alluded. More marked is the effect, when anxiety is added. This greatly reduces the functions of the stomach; it produces flatulency, and often diarrhœa; it sometimes affects even the kidneys; it almost always, when long-continued, produces permanent disease of the liver. Cancer of the stomach, moreover, and other malignant diseases, occur most frequently among the victims of mental depression and care." 85.

It is in vain that we tell men to divest themselves of care, and attend to their health. They cannot do it!

Our author makes many judicious observations on professional men and their avocations. Clergymen have alternations of study and exercise in the open air; but the latter is often on a very confined scale, their avocation preventing them from participating in sports or amusements.

"PRACTITIONERS of MEDICINE and SURGERY must next be noticed. Our office requires that a considerable portion of time be daily devoted to study, and the rest to professional visits. These, of course, afford exercise in the open air, and thus tend to invigorate the health,—while, on the contrary, the application of mind to study and research, tends to impair it. Night-calls are generally thought to be very injurious. I think the evil less than the public and the profession suppose; for, if we observe those who have for thirty or forty years been much engaged as accoucheurs, we shall find them as robust as others. Anxiety of mind does more, I conceive, to impair health, than breach of sleep, nocturnal exposure, or irregularity in meals. The body suffers from the mind. That sense of responsibility which every conscientious practitioner must feel,—the anxious zeal, which makes him throw his mind, and feelings, into cases of especial danger or difficulty,—break down the frame, change the face of hilarity to that of seriousness and care, and bring on premature age.* As a profession, we are by no means healthy. Indigestion is general, and diseases of the lungs and blood-vessels are frequent. I am aware that several instances of great age will be immediately remembered; but while referring to cases like these, we forget the number who die in middle age and youth. Inquiring occasionally after those whom I knew as students, I have been often surprised at the number of deaths. Among the youth of our profession, in an especial manner, is the mortality great. Pupils sent to distant medical schools at the end of their ap-

* "Ramazzini speaks very differently on the subject. He says that medical practitioners are comparatively exempt from ordinary diseases, in consequence of their good exercise, and their *hilarity* of mind, when they go home with their fees in their pockets,—'Dum bene nummati lares suos repetunt.' He adds, that medical men are never so unwell, as when no one else is unwell. The professor remarks, however, that they are subject to hernia from going up stairs, and catch dysentery from sitting besides their patients!"

prenticeship, and thus placed suddenly in a scene of dissipation, without governor or adviser,—mixing too with a large mass of youths similarly situated,—suffer from the evils and disease, which irregularity produces. While the steady youths, attending the hospitals, dissecting, hearing various lectures, and preparing for examination,—obliged, too, be it remembered, to acquire, in a couple of winters, that various knowledge which ought not to be acquired in triple the time,—are severely injured by the great application of mind. Hence, the students who come out of the lecture-room at the end of the session, we should scarcely recognize as the healthy young men, who entered it a few months before. Complaints of the stomach and bowels are common, and pulmonary consumption is by no means infrequent. The effects of wounds in dissection are well known to be very serious, and often fatal." 92.

Dr. Thackrah makes some pertinent remarks on education at Leeds. The children are crowded in rooms of disproportionate size, and the air is consequently contaminated. The "MARCH of INTELLECT" is too rapid —and the motions of the body are neglected. "Learning, or what is termed learning, absorbs the nervous energy which is necessary for the body." The young females are still worse off than the boys. It is vulgar to use the limbs as Nature designed—it is vulgar to take the food which Nature requires—and young ladies must not do any thing that is vulgar. Well! They must be contented to wave the vulgarity of health also.

"When girls leave school, the same system of muscular quietism is enforced. They must keep up their accomplishments by practice. Several hours a day they must devote to music, and frequently a considerable time to the more injurious occupation of drawing; most of the remaining day, they spend in finger occupations. Little time is devoted to exercise in the open air, and the exercise they *do* take is such as to chill, rather than invigorate the circulation. Need I urge that half the disorders of the young arise from the errors I have mentioned? Need I advert to remedies and preventives? They are obvious." 95.

Dr. T. adverts to the learned professions, whose sedentary avocations (Lawyers for example) are injurious to health. "Many of the legal profession indulge too much at the table; and almost all neglect the state of the bowels."

But here we must close our notice of Dr. Thackrah's work, which deserves the attention of the profession generally, and particularly of those residing in large cities and manufacturing districts.

